# Examination of the Urine

**Published:** 1904-11

**Authors:** 


Examination of the Urine. By G. A. deSantos Saxe, M.D., Pathologist to the Columbus Hospital, New York City. Duodecimo volume of 391 pages, fully illustrated, including 8 colored plates. Philadelphia, New York, London: W. B. Saunders & Company. 1904. (Flexible leather, $1.50 net.)

This is a new work on the subject of the examination of urine and it contains many features which are lacking in larger and more pretentious books on the subject. Saxe has written from a rather new viewpoint and while the book is a concise guide to a proper and satisfying examination none of the more important points have been overlooked or even slighted in the effort to be brief. One of the chief charms of the work is that it is not tiresome by repetition. Very little theory is considered. The purely theoretic matters are either lightly touched upon or ignored altogether, which is a decided relief and a saving of much paper and time. Unwieldy analytic points are briefly referred to only when absolutely necessary. The theory of urinary secretion and the methods of examination regarding the functional activity of the kidneys have been given more prominence than one usually finds in textbooks on the analysis of urine, and this in connection with the fact that Saxe does not use up otherwise valuable space in the exploitation of his own pet fads and fancies gives to the book an air of honest value which is refreshing. Special attention is paid to diagnosis and the interpretation of urinary findings with added hints as to methods of work based upon the author's own experience in the laboratory which are most helpful. Wherever weights and measures are given, the metric system is used except in rare cases in which the old system is employed in order to keep intact old formulas. Indicative of the scope of Saxe's work the first part of the book deals with the urine as a secretion; part two, the chemic examination of urine dealing with proteids, carbohydrates, urea and its congeners, the acetone group, coloring matter and inorganic constituents; part three is devoted to the microscope and is painstaking in its instruction and advice; the diagnosis of urinary diseases is taken up in part four and includes the diagnosis of functional efficiency of the kidney, cryoscopy, which is very excellently presented in detail; so much so that it is clear and intelligent; the electric conductivity of urine, the rate of excretion, the chemic activity of the kidney, cryoscopy of the blood as compared with that of urine and the toxicity of urine. The book is completed by a list of working reagents and apparatus and taken as a whole it is one of the most understandable works on the subject yet printed. N. W. W.

